# Thiamine Pyrophosphate Effects on Newborn Piglets as a Measure of Vitality and Survival Indicators

**DOI:** 10.3390/ani15050619

**Published:** 2025-02-20

**Authors:** Paloma Islas-Fabila, Herlinda Bonilla-Jaime, Patricia Roldán-Santiago, Luis Alberto de la Cruz-Cruz, Ofelia Limón-Morales, Carlos Antonio Jiménez-Collado, Héctor Orozco-Gregorio

**Affiliations:** 1 Programa de Doctorado en Ciencias Biológicas y de la Salud, Universidad Autónoma Metropolitana, Ciudad de México 09340, Mexico; paloma_islas@hotmail.com; 2 Departamento de Biología de la Reproductiva, Universidad Autónoma Metropolitana, Ciudad de México 09340, Mexico; bjh@xanum.uam.mx (H.B.-J.); ofelia.limon@yahoo.com (O.L.-M.); 3 Departamento de Reproducción, Facultad de Medicina Veterinaria y Zootecnia, Universidad Nacional Autónoma de México, Avenida Universitaria, Ciudad de México 04510, Mexico; patriciaroldan@fmvz.unam.mx; 4 Facultad de Medicina Veterinaria y Zootecnia, Universidad del Valle de Mexico-Coyoacán, Calzada de Tlalpan, Ciudad de México 04910, Mexico; ladelacruzc@hotmail.com; 5 Preservación del Bienestar Animal/Manejo de Fauna Silvestre, Departamento de Producción Agrícola, Universidad Autónoma Metropolitana, Calzada del Hueso 1100, Coapa, Villa Quietud, Coyoacán, Ciudad de México 04960, Mexico; 6 Laboratorios Manuell S.A., Jarciería 237, Morelos, Venustiano Carranza, Ciudad de México 15270, Mexico; dirmed@manuell.com; 7 Ingeniería en Producción Animal, Universidad Politécnica de Francisco I. Madero (UPFIM), Tepatepec 42660, Mexico

**Keywords:** asphyxia, piglets, thiamine pyrophosphate, behavior, vitality

## Abstract

The objective of this study was to evaluate the prophylactic effect of thiamine pyrophosphate (TPP) on the vitality scores of piglets based on their behavior and survival. A total of 149 piglets born from 15 multiparous sows were evaluated. The sows were randomly divided into two groups, control and TPP, with treatments administered 24 and 12 h before the expected farrowing date. The duration of farrowing was recorded. Furthermore, for all newborns, the Apgar vitality scale, teat suckling, newborn weight and weight at weaning, piglet vitality based on behavior (at birth and at 24 h), and skin temperature (at birth and at 24 h) were evaluated. The results indicated that the sows treated with TPP presented a lower farrowing duration (*p* = 0.0060) and their piglets exhibited a higher percentage in the piglet vitality-based behavior score (>50%). In addition, the newborn piglets of TPP-treated sows, which exhibited higher scores in behavior parameters, also displayed higher scores (>80%) in the Apgar vitality scale (>8), suckled on the teat for longer periods of time, and had higher daily weight gain (*p* < 0.0001). Our findings suggest that administering TPP at the end of gestation may shorten labor while also increasing the vitality of newborns. Therefore, it could be considered that, in practice, the administration of this treatment could have an impact on the energy that sows need during the farrowing process for the initiation of uterine contractions and abdominal effort. Therefore, this treatment could have an impact on the productivity and well-being of sows with a history of dystocic farrowing, which can increase the incidence of endometritis, vulvar discharge, placental retention, or mastitis–metritis–agalactia syndrome—alterations that can result in the reduced growth of piglets and a higher mortality before weaning. Therefore, the application of this treatment could not only reduce the probability of sows presenting these problems but perhaps also increase the probability of their offspring surviving in the first days after birth.

## Simple Summary

During the expulsion phase and birth of piglets, there are diverse stress factors that may reduce the vitality of the newborn piglets and can make piglets less adaptable to life outside the uterus by presenting greater physiological, metabolic, and behavioral alterations. For these reasons, diverse studies have focused on developing protocols that increase the newborn’s vitality. These investigations were mainly based on strategies focused on administering energetic supplements. In this regard, a recent study observed that the prophylactic administration of thiamine pyrophosphate (TPP) to sows at the end of gestation can increase the percentage of neonates with high vitality. To investigate this, the objective of this study was to evaluate the prophylactic effect of TPP on the vitality scores of piglets based on their behavior and survival. The results showed that prophylactic administration of TPP to sows at the end of gestation increased the percentage of neonates, with higher scores in the parameters of movement ability and number of completed circles around the pen. Furthermore, the newborn piglets of TPP-treated sows presented higher clinical vitality scores, suckled the teat for longer periods, and obtained a higher weight gain at 21 days.

## 1. Introduction

During the expulsion phase and birth of piglets, there are diverse stress factors (environmental, physiological, ethological, amongst others) that may affect the vitality of the newborn piglets [1,2]. Vitality is an important indicator at birth as it correlates with the growth rate (r^2^ = 0.35; *p* < 0.001) [3] and mortality rate (r = −0.65; *p* < 0.05) [4] (survival rate at 7 days and at 10 days of life) [3]. Additionally, it has been observed that piglets that presented a lower vitality at birth also presented less capacity to adapt to life outside the womb [4] as they exhibited higher physiological, metabolic, and behavioral alterations. For example, piglets with low vitality have a lower intake of colostrum (135 g) as well as lower weights at birth (approx. 300 g less; *p* < 0.01) and at weaning (approx. 1.15 kg less; *p* < 0.01), in comparison to neonates with high vitality [5]. Similarly, Sadeghi et al. [6] mentioned that neonates with low vitality exhibit less movement, lower heart and respiratory rates, deformities, and divergent skin color. Equally, major breakage incidence in the umbilical cord (75.68%; *p* < 0.0001), lower levels of pO_2_ (21.7 mmHg), and higher-than-normal glucose levels (83.5 mg/dL) and lactate (90 mg/dL) associated with perinatal asphyxia have been observed [7]. For these reasons, diverse studies [3,4,8,9,10] have assessed various physiological variables (obtained after recording the piglets’ heart rate, muscle tone, onset of respiration, and attempts to stand at birth) and behavioral parameters (e.g., ability to first suckle after birth or capacity to perform rooting behavior using a neurobehavioral test or rooting-response test) that reflect the newborn’s vitality [2]. Other investigations have focused on developing protocols that increase the newborn’s vitality and decrease physiological, metabolic, and behavioral alterations exhibited by low-vitality newborns. These investigations were mainly based on increasing the vitality of the newborn piglets by means of strategies centered on administering energetic supplements directly to the newborn [11,12,13] or to the mother during gestation [10,14].

Supplementing energy after birth provides support for the newborn piglets to cover the higher energetic usage demand associated with their low thermoregulatory capacity; this low capacity is due to a lack of brown adipose tissue, low glycogen reserves, and a scarce hair layer, which impede the conservation of heat and cause the piglets to rely almost exclusively on shivering thermogenesis [15,16]. Decreased skin temperatures in newborn piglets result in a lower intake of colostrum, an inadequate stage of immunological protection [16], and greater alterations in gas exchange, acid–base balance, and energy metabolism [8,17].

Therefore, an additional option could consist of applying molecules associated with the production of energy to preserve the energetic metabolism in the uterus, acting on enzymatic pathways before the birth of the piglets [18]. In this regard, in a study [19] in neonatal rats (11-day-old) with experimentally induced hypoxia, it was found—histologically—that animals that received TPP one hour before suffering hypoxia exhibited less brain damage in the motor cortex, somatosensory cortex, and striated cortex in comparison with the control group. Likewise, it is important to note that, in this same study, it was shown that neonates who are administered TPP after hypoxia exhibit tissue damage similar to animals in the control group. Therefore, these results suggest that once the cell death process has started, there is practically no possibility of reversing it. In the same way, a recent study [20] observed that the prophylactic administration of TPP to sows at the end of gestation can reduce the duration of farrowing (*p* = 0.0060), increase the percentage of neonates with high vitality (60%), and can cause a greater increase in weight at weaning (*p* < 0.0001). It should be noted that these findings should be interpreted with caution as in this experiment, all sows were induced to farrow with prostaglandins, which may have had an effect on the duration of farrowing.

On the other hand, it is important to note that in recent years TPP has been shown to have different protective effects, for example, one study indicates [21] that it can protect the brain and heart tissue against oxidative and inflammatory damage because it can prevent the increase in levels of tumor necrosis factor alpha (TNF-α), interleukin 1β (IL-1β), and interleukin-6 (IL-6) in brain and heart tissues. In addition, it has been shown that TPP could be beneficial in the treatment of cyclophosphamide-induced ovarian injury and infertility [22] since the administration of 25 mg/kg of TPP intraperitoneally in the anterior abdomen in rats (one dose) decreases the levels of the oxidant malondialdehyde (MDA), proinflammatory nuclear factor kappa B (NF-κB), tumor necrosis factor alpha (TNF-α), and interleukin 1 beta (IL-1β); in addition, it reduces severe histopathological damage associated with cyclophosphamide in ovarian tissue [22]. In this same line of research, a protective effect of TPP has been demonstrated by improving cisplatin-induced testicular damage in rats [23]. This depends on the dose since doses of 100 mg/kg of TPP increase the protective effects compared to a lower dose (50 mg/kg for 5 consecutive days). This protective effect of TPP can perhaps be attributed to its antioxidant and antiapoptotic actions. On the other hand, Mahdavifard and Nakhjavani [24] demonstrated that TPP can reduce the main risk factors for diabetic complications and correct alterations in glucose and lipid metabolism. This is because it has a reducing effect on glycation, oxidative stress, and inflammation markers; it also exerts an effect on insulin action, glyoxalase system activity, and the lipid profile in diabetic rats. Therefore, it is recommended for the treatment of diabetes.

Despite previous studies [18,19,20] demonstrating that TPP has beneficial effects if administered before a hypoxic event, it is rather complicated to determine in advance whether an organism will be exposed to an event of this nature or not in the neonatal phase. Regardless, valuing the potential of TPP in affecting behavior—as a measure of vitality—and the main survival indicators of low-vitality newborns is crucial [20].

Finally, it is important to point out that, to the best of our knowledge, the present study is one of the first to assess the effect of TPP administration at the end of gestation on the behavior—as a measure of vitality—and the survival indicators of newborn piglets.

## 2. Materials and Methods

### 2.1. Animal Care

All experimental procedures were approved by the Research Committee of the Universidad Autónoma Metropolitana (CBS.310.18) and the Animal Care and Use Committee of Universidad Autónoma Metropolitana–Iztapalapa, in accordance with the current Mexican laws of animal protection (Protocol # 062).

### 2.2. Animals and Housing Conditions

This study was carried out in the maternity area of a commercial pig farm located in the northeast of Mexico City and included 149 neonatal piglets born from 15 multiparous (from second to fifth parity; weight: 200–280 kg) Yorkshire–Landrace sows. The sows had an average weaning–estrus interval of 5 ± 1.5 days and were artificially inseminated: the first insemination was carried out 12 h after detecting estrus (which was verified by direct contact with a mature boar and a back-pressure test), and a second insemination was repeated 12 h after carrying out the first insemination. With the objective of minimizing variations in insemination operations, all inseminations were performed by an experienced technician. The day of the first insemination was considered day 0 of gestation. After insemination and until day 107 of gestation, sows were housed in groups of nine in pens that measured 6.7 m × 4.4 m (3.3 m^2^ per sow). Each group was fed in nine individual feeding stalls (1.8 m × 0.55 m) two times per day—morning and afternoon—and water was supplied ad libitum through nipple drinkers. The sows were fed 1.8 kg/d of the gestation diet from day 1 to 30, 2.4 kg/d from day 31 to 90, and 3.0 kg/d from day 91 to 113 of gestation, approximately. On day 107 of gestation, the sows were moved to a climate-controlled (temperature of 23 ± 2 °C and 60% relative humidity) farrowing room and housed in individual farrowing crates (1.45 m long × 0.8 m wide × 0.57 m high). Likewise, it is important to note that sows entered the farrowing room with a backfat thickness > 17 and <19 mm according to the criteria indicated by Mun et al. [25] and with a body condition score of 3 to 3.5 because, at the end of lactation, it was expected that the sows would have a score of 3 to 2.5 at least.

The gestation diet was made up of corn (80%), soybean meal (16%), and premix (4%) that provided 3176.64 kcal/kg of metabolizable energy that was 13.86% protein, 2.25% crude fiber, 0.64% digestible lysine, 0.24% digestible methionine, 0.54% digestible threonine, 0.71% calcium, 0.39% phosphorus, and 0.18% sodium, following the standard levels of vitamin requirements corresponding to the recommendations of Santos et al. [26]. However, the feed offered to the sows was reduced to 2.0 kg one day before the expected farrowing date. During gestation and lactation, sows had ad libitum access to water. On the other hand, during lactation, sows were fed a commercial lactation diet [14.5 MJ digestible energy (DE)/kg, 19% crude protein (CP), 1.2% total lysine (Lys)] and received 2 kg/day of feed on day 0 of lactation. Subsequently, the amount of feed was increased from day 2 to day 10 of lactation (0.5 kg/day until reaching 6 kg/day). During the last days of lactation (11 to 21), the amount of feed provided was 6.5 kg/day. It is also important to note that one of the feeding strategies that was carried out as a supplement to breast milk in piglets was the administration of a pre-starter (protein 20%, fat 5%, fiber 5%, ashes 10%, moisture 13%) from day 12 of life until the day of weaning.

### 2.3. Treatments

The applied exclusion criteria were as follows: Sows with a duration of expulsion > 300 min [27] that required obstetric handling due to a delay longer than 90 min between the expulsion of piglets or required the application of oxytocin before their contractions ended. Sows that were extremely nervous or presented a level of dorsal fat ≥ 28 mm of back fat at farrowing were also excluded, in accordance with the methodology outlined by González-Lozano et al. [28]. Likewise, it is important to mention that, in the control group, a total of 9 sows were evaluated; however, 3 sows were excluded from this study according to the exclusion criteria. In addition to this, not all of the newborns from the 15 sows were evaluated as those who were born after the application of oxytocin or went through obstetric management were not taken into account in this study, with the objective of not intervening with the treatment.

**Figure 1:**
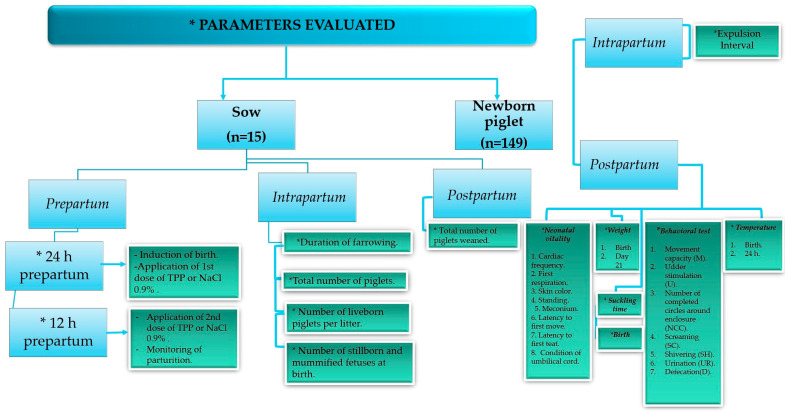
Description of the parameters evaluated in sows treated with and without TPP and in their piglets.

The sows were randomly distributed into two different treatment groups: Group (1) control (*n* = 6): sows served as negative controls in a placebo group and were treated with 0.9% NaCl (25 mL CS PiSA^®^ 0.9%, PiSA Laboratories, Reforma 180, Juárez, Cuauhtémoc, 06600 Ciudad de México, CDMX); Group (2) TPP (*n* = 9): sows received 10 g of cocarboxylase diluted in 25 mL of 0.9% NaCl (Carzilase^®^, Manuell Laboratories, Mexico City, Mexico). The dose of TPP used was the maximum proposed by the manufacturer. To guarantee bioavailability at all times of farrowing, TPP (Carzilase^®^) was administered 24 and 12 h before the probable date of farrowing. The route of administration was intramuscular (i.m.) into the neck region. All births were induced 24 h prior to the probable date of farrowing (day 114) with 10 mg (i.m.) of prostaglandins F2α (Dinoprost Tromethamine, Lutalyse^®^; Pharmacia & Upjohn, Mexico City, Mexico). Monitoring of births began 12 h after the injection of prostaglandins (Figure 1).

### 2.4. Reproductive Variables of Sows

The farrowing duration (time between the first and last piglets born), total number of piglets born per litter, number of piglets born alive per litter, stillbirths and mummified fetuses at birth, and number of piglets at weaning were evaluated.

### 2.5. Modified Apgar Vitality Scale

Neonatal vitality was assessed immediately after birth in all piglets using the scale described by Revermann et al. [29] and Sánchez-Salcedo et al. [30]; time interval from birth to first breath, heart rate, snout skin color, meconium staining of the skin, and time interval from birth to first standing were evaluated according to Sánchez-Salcedo et al. [30]; latency at the first move, latency to first teat contact, and the condition of the umbilical cord were evaluated according to the methodology of Revermann et al. [29]. Each parameter was classified into 3 categories: 0 (the worst), 1 (intermediate), and 2 (the best). The parameters were then used to obtain an overall vitality score of 0–10 for each piglet. To estimate the vitality score, the total amount of points was added to each of the 8 parameters evaluated and the vitality average was calculated, considering 16 points to be equivalent to a score of 10.

The evaluated parameters were as follows: (a) time interval from birth to first breath was taken into account when chest movements accompanied with exhalation of air were observed (0 = >1 min, 1 = 16 s to 1 min, and 3 = <15 s); (b) heart rate (0 = <120, 1 = 121–160, and 2 = >161 beats/min); (c) snout skin color (0 = pale, 1 = cyanotic, and 3 = pink); (d) meconium staining of the skin (0 = absent, when there is no evidence of meconium staining; 1 = moderate, when meconium covers less than 50% of the body surface; and 2 = severe, when meconium covers more than 50% of the body surface); (e) time interval from birth to first standing (0 = >5 min, 1 = 1–5 min, and 2 = <1 min); (f) latency at first move (0 = no movement within 15 s, 1 = within 15 s a little movement [within 15 s the piglet made circular head movements or showed searching behavior but was unable to turn the axis of its body > 90° from its initial orientation at least once], and 2 = within 15 s a lot of movement [within 15 s the piglet showed circular head movements or searching behavior and was able to turn the axis of its body > 90° from its initial orientation at least once]) [3,29]; (g) latency to first teat contact—the interval between birth and the first time the newborn made contact with the mother’s teat (0 = after 30 min, 1 = 10–30 min, 2 = within 10 min); (h) condition of umbilical cord (0 = ruptured < 15 cm, 1 = ruptured ≥ 15 cm, 2 = connected). Heart rate and blood oxygen saturation were determined using a pulse oximeter (Edan Instruments Inc., Nanshan, Shenzhen, China).

Likewise, before evaluating vitality, the expulsion interval (s) was recorded, which was defined as the time elapsed between the expulsion of one piglet and the next. In addition, at the end of the vitality assessment, the weight at birth (g) of each piglet was recorded and piglets were weighed again weekly until the end of lactation (21 d). Body weight was recorded using a digital scale that is precise to 0.1 kg (Salter Weight-Tronix Ltd., West Bromwich, UK). Immediately after evaluating the weight at birth, piglets were individually identified according to their birth order. At the end of marking, their sex was recorded, and the piglets were placed next to the vulvar region of their sows in order to evaluate the suckling time—which started when the piglet had a teat in its mouth and was actively suckling and ended when the piglet stopped being active on the udder—according to Illmann et al. [31].

### 2.6. Piglet Vitality Based on Behavior

**Figure 2:**
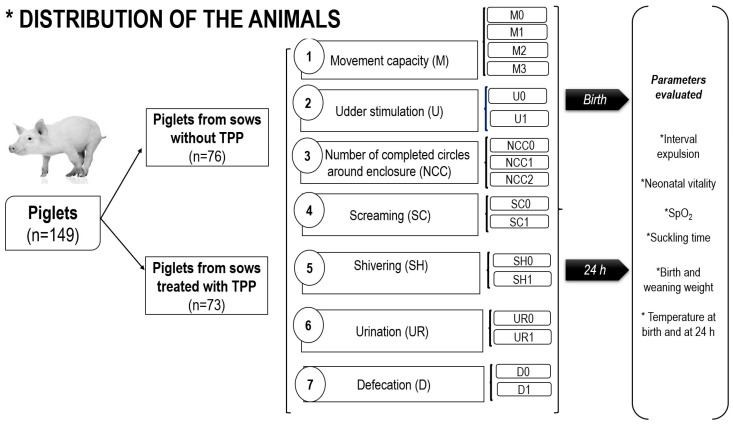
Distribution of the animals. *n* = number of piglets sampled. Piglets born to sows treated with and without TPP were classified according to the vitality score (0, 1, 2, and 3) obtained in each of the categories used within each behavioral parameter (M, U, NCC, SC, SH, UR, and D), which were evaluated at birth and at 24 h. In both groups evaluated at birth and at 24 h, the effects of the expulsion interval, neonatal vitality, SpO_2_, suckling time, birth and weaning weight, and temperature at birth and at 24 h were evaluated.

Vitality was evaluated based on the behavior of the piglets (Table 1) after their first suckling and at 24 h of life. This evaluation was performed according to Muns et al. [3] and included the assessment of four parameters: (1) capacity of movement (M); (2) teat stimulation (U); (3) number of circles completed around the yard (NCC); and (4) screaming (SC). Likewise, other parameters were added, including the following: (5) shivering (SH), as described by Tuchscherer et al. [32]; (6) urination (UR); and (7) defecation (D), as described by Lürzel et al. [33] (see Table 1). Each piglet received a vitality score (of 0, 1, 2, or 3), in accordance with the categories utilized within each behavior parameter (Figure 2).

As the piglets were assessed individually, they were separated from the litter and introduced into a pen with a diameter of 80 cm × 60 cm height, with solid ground and plastic walls, and which was open both in the inferior and superior areas. The yard where all evaluations were conducted was situated in the corridor, on solid ground, and opposite the birth cage of each sow. Videotapes of each piglet with a duration of 30 s were captured in the delimited area (the video camera was a Sony Handy Cam HDR-SR5, placed on a Manfrotto Autopole at a height of 2–2.5 m, and the video resolution was 1440 × 1080 pixels). Afterward, each tape was analyzed on a portable laptop to estimate the angles of the movement included in all parameters, where each quadrant was divided into angles of 0 ° to 360 °. Five repetitions were carried out for each videotape.

### 2.7. Temperature Evaluation

Skin temperature was assessed with an infrared laser thermometer (Penrui JRT200^®^, Items Industriales, S.A. Carr. a El Salvador, Cdad. de Guatemala, Guatemala), recorded at birth and when the neonates were 24 h old. The thermometer was placed approximately 3 cm from the piglet’s head. To reduce the variability in the measurements, three temperature data points were taken according to Schmid et al. [34]—who indicate that repeated measurements with an infrared thermometer taken on an animal’s head do not substantially differ.

### 2.8. Statistical Analysis

Descriptive analysis was initially carried out, and data are reported as frequencies for all behavior parameters: movement capacity (M), udder stimulation (U), number of completed circles around the enclosure (NCC), screaming (SC), shivering (SH), urination (UR), and defecation (D).

Residual plots were used to check model assumptions (e.g., normality). Total stillborn piglets and SpO_2_ (%) at birth were non-normally distributed and the data were transformed using a base-10 logarithm and were presented as means of least squares (GLIMMIX). Subsequently, a completely random design was used. Continuous data—interval of expulsion (min), vitality score at birth (modified Apgar), SpO_2_ (%) at birth, suckling time (s), weight (g), and temperature (°C)—are presented as mean ± SD and were compared between the two groups (neonates with and without TPP) by means of an ANOVA test under the GLM (General Linear Models) procedure of the statistical program JMP 8.0 (JMP Institute, Marlow, Buckinghamshire, UK). Post hoc comparisons between the groups were assessed using 95% confidence intervals for the difference in the means of least squares and *p*-values (two-tailed significance) for overall differences and specific treatment differences. Results were presented using means of least squares and the standard error of the means (SEM). Finally, multiple comparisons of means were performed using Tukey’s T test. Multiple comparisons of means were carried out using Tukey’s test. The level of significance was set at *p* < 0.05. Piglet mortality (stillbirth and mummies) was analyzed using the Chi-square test.

## 3. Results

### 3.1. Reproductive Variables in Sows

The sows of the control group presented a higher farrowing duration in comparison to sows treated with TPP (198.66 vs. 150.11 min, respectively; *p* = 0.0060) (Table 2). However, there were no significant differences in the total number of piglets per litter, the live neonate piglets by litter, and the number of piglets weaned per litter according to the administration of TPP (*p* > 0.05). Additionally, there was a lower number of stillbirths in the litters of sows treated with TPP (0.8 vs. 0.375 stillborn piglets, respectively).

### 3.2. Piglet Vitality Based on Behavior

**Figure 3:**
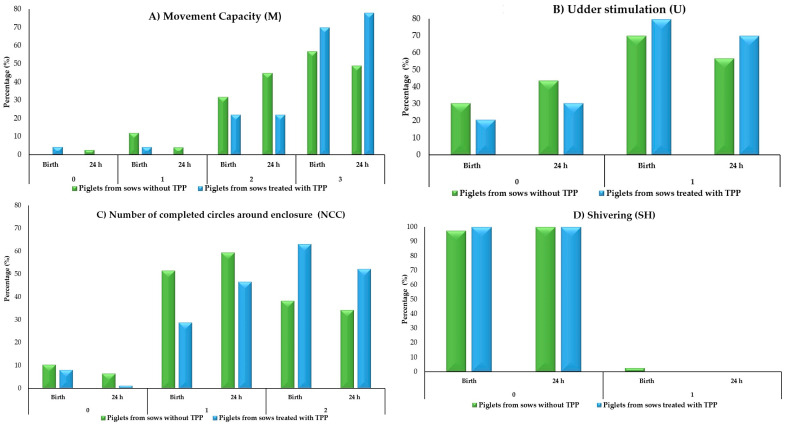
(**A**–**D**) Piglet vitality based on behavior: Regarding the results of the behavioral evaluation at birth and at 24 h, a greater percentage (>50%) of piglets born to sows treated with TPP obtained high scores in the parameters of movement capacity (M3) and teat stimulation (U1). Likewise, these piglets were able to turn their body axis 360° from their initial orientation or walk along the pen boundaries at least twice (NCC2) and, similarly, >80% of piglets born to TPP-treated sows did not show a tremor response (SH0) at birth and at 24 h.

Table 3 shows the distribution of scores obtained for the piglets born from sows treated with and without TPP for each behavior parameter, evaluated at birth and at 24 h (M, U, NCC, SC, SH, UR, and D) (Figure 3).

#### 3.2.1. Movement Capacity (M)

**Figure 4:**
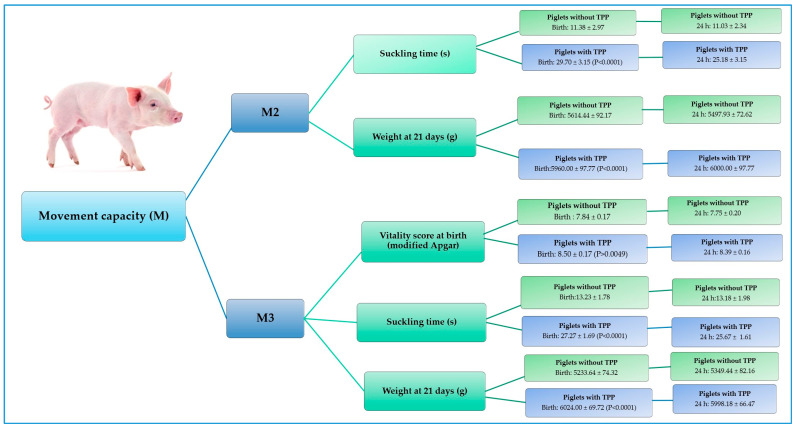
Effect of thiamine pyrophosphate on modified Apgar vitality, suckling time, and weight according to the movement capacity parameter (M) evaluated at birth and at 24 h in neonatal piglets. This figure summarizes the parameters for which a significant effect of TPP was observed in piglets that exhibited higher scores in movement capacity (M2 and M3).

In the present study, it was observed that piglets from the TPP group presented significantly higher movement capacity at birth and at 24 h. The changes in the following variables were evident: the modified Apgar vitality score, time of suckling at birth, weight, and temperature (Table 4). In regards to the modified Apgar vitality score, it was observed that the piglets born from sows treated with TPP that were able to move rapidly (M3) at birth obtained higher scores (>8; *p* = 0.0049) and presented higher weight gain at day 21 (790 g; *p* < 0.0001) when compared to the neonates born from control group sows (Table 4 and Figure 4). Additionally, in terms of the suckling time variable, it was observed that the piglets of sows treated with TPP that were able to move slowly (M2) and quickly (M3)—both at birth and at 24 h after their birth—suckled on the mother’s teat for longer periods (25.18 and 29.70 s, respectively; *p* < 0.0001) in comparison to the neonates of the control group sows at both times.

In contrast, the piglets born from sows treated with TPP that were able to maintain a voluntary position but were incapable of moving (M1) at birth showed a 1.53 °C higher temperature at 24 h when compared to the neonates of control sows (*p* < 0.0001). Similarly, in the piglets born from sows treated with TPP that were able to move slowly (M2) at 24 h, a significant increase (0.54 °C) was observed in their temperature at 24 h when compared to the piglets born from control sows (*p* < 0.0001; Table 4).

#### 3.2.2. Udder Stimulation (U)

**Figure 5:**
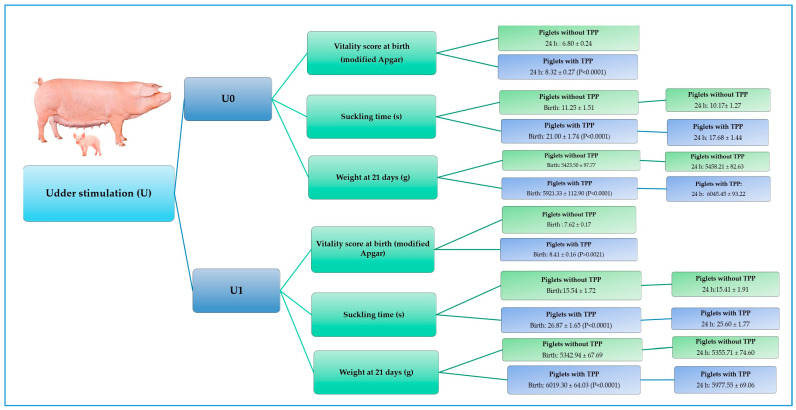
Effect of thiamine pyrophosphate on modified Apgar vitality, suckling time, and weight according to the udder stimulation parameter (U) evaluated at birth and at 24 h in neonatal piglets. This figure summarizes the parameters for which a significant effect of TPP was observed in piglets that exhibited low and high scores in udder stimulation (U0 and U1).

In the parameter of udder stimulation, significant changes were observed with the administration of TPP, mainly in the piglets that did not evidence udder stimulation movements or searching behavior within 30 s (U0; see Table 5). Piglets born from control sows that did not demonstrate udder stimulation behavior (U0) at birth and at 24 h presented a longer expulsion interval (11.37 and 10.51 min, respectively; *p* < 0.0222; see Table 5 and Figure 5). Likewise, the piglets of the same control group that did not manifest teat stimulation movements (U0) presented lower scores of clinical vitality at birth and at 24 h (6.51 and 6.80, respectively; *p* < 0.0001).

Additionally, they dedicated less time to suckling their mother’s teat, both at birth and at 24 h (9.75 s and 7.51 s, respectively; *p* < 0.0001) when compared to piglets born from sows treated with TPP (Table 5).

On the other hand, in terms of weight and temperature (Table 5), a significant effect of TPP (*p* < 0.0001) was observed in piglets born from sows treated with TPP that did not show teat stimulation movements and searching behavior (U0) at birth and 24 h—they weighed 497.83 and 587.24 g more at 21 days of age. Similarly, the neonates of this group (TPP) that showed no udder stimulation movements and searching behavior (U0) had a higher temperature at 24 h when compared to the piglets born from control sows. These piglets were better able to walk along the pen boundaries at least once (NCC1) or twice (NCC2) within 30 s and, at 24 h, presented high scores (>8) on the clinical vitality scale compared to the control sows (piglets with TPP: 38.41 ± 0.12 °C vs. piglets without TPP: 37.86 ± 0.11 °C; *p* < 0.0001).

#### 3.2.3. Number of Completed Circles Around Enclosure (NCC)

**Figure 6:**
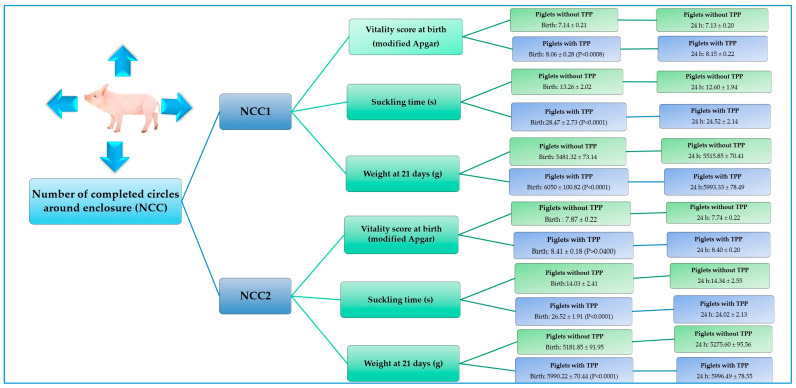
Effect of thiamine pyrophosphate on modified Apgar vitality, suckling time, and weight according to the number of completed circles around enclosure parameter (NCC), evaluated at birth and at 24 h in neonatal piglets. This figure summarizes the parameters for which a significant effect of TPP was observed in piglets that exhibited higher scores in number of completed circles around enclosure (NCC1 and NCC2).

Significant changes were observed in this behavioral variable regarding the effect of TPP and/or the effect of vitality score and suckling time at birth (Table 6). Piglets born from sows treated with TPP were able to turn their body axis 360° from their initial position more (*p* < 0.0008 and NCC2: *p* < 0.0400) and had longer teat-suckling periods (>24 s; NCC1: *p* < 0.0001 and NCC2: *p* < 0.0001) when compared to piglets born from control sows (Figure 6). On the other hand, with respect to weight, we observed an effect of TPP administration when neonates exhibited a higher score (1–2) in this parameter: piglets born from control sows that were able to rotate their body axis 360° from their initial orientation or walk along the limits of the bucket once within 30 s (NCC1) or at least twice within 30 s (NCC2) at birth and at 24 h presented 569.18 (NCC1 at birth: *p* < 0.0001), 808.37 (NCC2 at birth: *p* < 0.0001), 477 (NCC1 at 24 h: *p* < 0.0001), and 720.89 (NCC2 at 24 h: *p* < 0.0001) grams lower weight at 21 days of age when compared to piglets born from TPP-treated sows (Table 6).

#### 3.2.4. Screaming (SC)

In this indicator, the clinical vitality score, suckling time, weight, and temperature (Table 7) presented changes due to the effect of TPP administration at the end of gestation. It was observed that piglets born from control sows that did not show vocalizations during the observation time (SC0) at birth and at 24 h presented 1.44- and 1.25-lower scores in the modified Apgar vitality scale, respectively, and spent 15.42 and 18.07 s less time suckling the mother’s teat, respectively, when compared to piglets from sows treated with TPP (clinical vitality: *p* < 0.0001; suckling at birth: *p* < 0.0001). In addition, piglets born from sows in the control group that did not show (SC0) or showed (SC1) vocalizations during the manipulation or observation time at birth and at 24 h presented lower weight at 21 d; namely, 578.43 g (SC0 at birth: *p* < 0.0001), 678.25 g (SC1 at birth: *p* < 0.0001), 721.71 g (SC0 at 24 h: *p* < 0.0001), and 522.23 g (SC1 at 24 h: *p* < 0.0001) less than that of piglets born from TPP-treated sows. Finally, piglets born from sows in the control group that manifested vocalizations (SC1) during handling or observation time at 24 h presented a 0.41 °C lower temperature at 24 h (*p* < 0.0001) compared to neonates from sows in the control group.

#### 3.2.5. Shivering (SH)

Regarding this variable, significant changes were observed in the group of piglets born from TPP-treated sows that did not manifest trembling behavior (SH0) at birth and at 24 h, with these neonates presenting higher scores (>8) on the clinical vitality scale (*p* < 0.0001), longer suckling times (>23.59 s; *p* < 0.0001), and a higher weight gain (639 and 601 g more weight, respectively; *p* < 0.0001) at day 21 (Table 8) in contrast to piglets born from control sows. Regarding temperature (Table 8), neonates in all groups that did not manifest tremor behavior at birth (SH0) and 24 h presented a skin temperature increase of >1.7 °C (*p* < 0.0001) from birth to 24 h of age.

#### 3.2.6. Urination (UR)

It was observed that piglets born from sows treated with TPP that did not show urination behavior (UR0) at birth and at 24 h showed a higher clinical vitality score (*p* < 0.0001) and suckled their mother’s teat for longer when compared to the control group of piglets (*p* < 0.0001; Table 9). Likewise, piglets with this UR0 condition and with TPP presented higher weight gain at day 21 (birth: 626.08 and 24 h: 589.98 g, respectively; *p* < 0.0001; Table 9).

Regarding temperature (Table 9), a significant effect of time was observed among the different groups as—regardless of whether piglets were born from sows treated with TPP or not—neonates in all groups that did not exhibit urination behavior (UR0) at birth and at 24 h showed a >1.7 °C increase (*p* < 0.0001) in their skin temperature from birth to 24 h of age.

#### 3.2.7. Defecation (D)

Significant changes due to the effect of TPP and no defecation (D0) at birth and 24 h were observed in the following variables: clinical vitality score, suckling time at birth, weight, and temperature (Table 10). Piglets born from TPP-treated sows that did not exhibit defecation behavior (D0) at birth and at 24 h presented higher scores on the clinical vitality scale (>8; *p* < 0.0001) and had longer teat suckling times (>20 s; *p* < 0.0001; Table 10). In addition, they also presented a higher weight gain at 21 days (birth: 636.07 g and 24 h: 598.39 g more weight; *p* < 0.0001), in contrast to piglets born from control sows (Table 10). On the other hand, in the temperature variable, time had a significant effect among the different groups: in the piglets of all groups (i.e., independently of the administration of TPP) that did not present defecation behavior at birth and at 24 h, an >1.8 °C increase (*p* < 0.0001) in their skin temperature was observed from birth to 24 h of age (Table 10).

## 4. Discussion

### 4.1. Reproductive Variables in Sows

Our results suggest that the shorter farrowing duration due to TPP administration in sows prior to farrowing was probably associated with the fact that thiamine—being an essential cofactor for ATP generation [35]—possibly provided a greater supply of energy to the uterus during the farrowing process, resulting in a shorter farrowing duration [36]. In this sense, it is known that sows that experience shorter farrowing duration present a higher plasma glucose concentration (*p* < 0.05) as the energy state of sows has a remarkable influence on their farrowing kinetics [36,37], where a low energy state during labor can decrease uterine contractions [36].

On the other hand, a shorter duration of farrowing in sows with TPP is probably associated with neonates exhibiting higher vitality scores and weight gain at weaning since, according to Tucker et al. [38], prolonged farrowing may increase the incidence of intrapartum hypoxia, which could be associated with brain damage and negatively affects vitality, growth, and survival during lactation [39].

It is important to mention that although statistically significant differences were observed for the effect of treatment on the duration of farrowing, these results should be interpreted with caution as the farm where the experiment was conducted is used as part of a strategy to improve neonatal survival—inducing farrowing by the administration of prostaglandin F2α (PGF2α). Therefore, all sows were induced to farrow and it is likely that the administration of this hormone had an effect on triggering farrowing by inhibiting progesterone secretion and perhaps also had an effect on the stimulation and coordination of myometrial activity—through the release of calcium from the endoplasmic reticulum, the opening of the calcium-dependent channels, or by stimulation or inhibition of the adenyl–cyclase system (producing relaxation as myometrial contraction) [40].

### 4.2. Piglet Vitality Based on Behavior

With respect to the results of the behavioral assessment at birth and at 24 h, a higher percentage (>50%) of piglets born from TPP-treated sows scored high in the following parameters: movement ability (M3), teat stimulation (U1), number of circles completed around the pen (NCC2), and vocalizations (SC1) at birth and at 24 h. Similarly, >80% of piglets born from TPP-treated sows did not manifest a trembling (SH0), urination (UR0), and/or defecation (D0) response at birth and at 24 h. Therefore, the administration of TPP at the end of gestation is considered to have had a positive effect on the neonates.

A possible explanation as to why most piglets from TPP-treated sows presented high scores in the parameters of movement ability (M3), teat stimulation (U1), and number of completed circles around the pen (NCC2) could be because in addition to the coenzymatic function of TPP in the metabolism, thiamine is also involved in the structure, stability, and function of membranes, including axoplasmic, mitochondrial, and synaptosome membranes [41]. In addition, it has been found that thiamine acts against the cytotoxicity induced by various agents, such as reactive oxygen species (ROS) or reactive nitrogen species (RNS) [42]. This could have contributed to making the behavioral response of piglets with TPP more efficient at birth.

In addition, it has been observed that the TPP can play a role in the regulation of cholinergic neurotransmission [43]. In the present study, this neurotransmission may have played a fundamental role in affecting the M, U, and NCC parameters since it is involved in sensorimotor integration since the cholinergic neurons in the basal prosencephalon project to several brain regions, including the visual and auditory cortex as well as the hippocampus and amygdala [36,44].

Coupled with this, cholinergic terminals in sensory cortices can transmit signals associated with the motor state; for example, the activity of the basal forebrain-derived cholinergic axons in the auditory cortex can be related to ongoing movements, including rhythmic exploratory movement, and can modulate auditory sensory responses [36,45]. The abovementioned is important since, in the present study, the thiamine may have influenced piglets born from TPP-treated sows to exhibit higher scores in the M, U, and NCC parameters at birth and at 24 h as these types of parameters require the integration of sensory, motor, auditory, and visual functions.

Additionally, although tremor behavior is considered a defense against acute exposure to cold, it is performed for the purpose of producing heat through skeletal muscle by means of the antagonistic groups of skeletal muscles (flexors and extensors) that are activated, and heat is generated by the hydrolysis of adenosine triphosphate (ATP) and transferred to the center of the body as heat [37,46]. In this study, regardless of TPP administration at the end of gestation, more than 80% of piglets born to sows treated with and without TPP did not show shivering behavior (SH0) at birth and at 24 h. This may be related to the weight of the neonates since the neonates of all groups at birth exhibited a weight > 1 kg; this could also perhaps be due to the management that the piglets underwent after birth since, in the farm where this study was carried out, the neonates of all groups underwent a drying routine and had access to a warming area with boxes placed under a heat source and with straw in which the piglets were confined for short periods after birth in order to minimize heat loss.

#### 4.2.1. M, U, NCC, and SH

In terms of the parameters M, U, NCC, and SH, it was observed that piglets born from sows treated with TPP that presented high scores in the M and NCC parameters, as well as low scores in U and SH, presented significantly higher scores in the modified Apgar vitality scale. Furthermore, these piglets suckled the teat for a longer time and exhibited higher weight gain at 21 days. According to Vanden Hole [47], a piglet can only survive past the first few days when it performs fundamental behaviors, such as standing up and moving toward its mother’s teat, or when it exhibits teat stimulation behavior while monitoring the mother’s position so that it can quickly move away if there is a risk of crushing.

Therefore, not only is piglet size an important factor for survival but locomotion can also be considered a key ecological function for neonatal survival, which involves cyclic movement patterns (characterized according to specific limb-to-limb and intra-limb coordination patterns) [48]. The fact that piglets born from the TPP group sows in this study presented high M and NCC scores may be associated with their better locomotion. In this regard, Islas-Fabila et al. [49] have pointed out that some parameters of the modified Apgar vitality scale—such as latency to connect with the mother’s teat—require that neonates integrate different olfactory and neuromuscular functions that allow for an oriented search for the mother’s teat and, therefore, ensure faster colostrum consumption.

The latency to standing up for the first movement is also a parameter that can be altered by locomotion. This could explain why a higher percentage of neonates from TPP-treated sows who exhibited high M and NCC parameter scores, as well as a low tremor parameter score (SH0), obtained high scores (>8) on the modified Apgar vitality scale. On the other hand, neonates of TPP-treated sows who exhibited high M and NCC parameter scores as well as a low tremor parameter score (SH0) not only showed high clinical vitality scores but also obtained a higher weight gain at 21 days. These results are in agreement with those of Uddin et al. [5] who observed that when neonates exhibit higher vitality, they consume more colostrum (135 g more colostrum at 24 h; *p* < 0.01) and, consequently, tend to have a higher weight gain at weaning (25 g; *p* < 0.01).

On the other hand, it is important to note that locomotion can be affected by birth weight [50,51]. Vanden Hole et al. [50] have noted that when neonates exhibit low birth weights (0.79 ± 0.17 kg), they exhibit reduced motor performance. The fact that piglets from TPP-treated sows exhibited birth weights > 1 kg likely contributed to their better locomotion and low tremor parameter score (SH0); thus, it is believed that coordinated movement patterns are not entirely innate—instead, in these precocial animals (e.g., pigs, which are able to move shortly after birth), rapid neuromotor maturation takes place, potentially as a result of reorganization or recombination of existing motor modules [48].

In addition, the fact that TPP neonates exhibited a low score in tremor behavior (SH0) and high scores in the M and NCC parameters—as well as in clinical vitality parameters—was possibly due to the role that thiamine plays in providing energy to nerve cells [52]. In this process, thiamine enables the different biochemical steps in the energy creation processes to occur through the pentose phosphate pathway, glycolysis, and Krebs cycle processes. These processes supply energy to the nerves, mainly in the form of adenosine triphosphate (ATP) or nicotinamide adenine dinucleotide phosphate (NADPH). Considering the above, thiamine is indirectly needed for the synthesis of nucleic acids, neurotransmitters, and myelin [35,52]. Therefore, a thiamine deficit can cause psychomotor retardation as some sensitive areas of the brain, such as the thalamus and mammillary bodies (part of the hypothalamus), are damaged by thiamine deficiency [35].

On the other hand, it is possible that the better locomotion of piglets born from TPP-treated sows (groups: M2, M3, NCC1, NCC2, and SH0 at birth and at 24 h) may have influenced their ability to claim a nipple and to connect with and suckle from their mother’s teat for a longer time without being crushed during the suckling process [50]. All of these actions require neonates to show neuromotor maturation given that connecting to the mother’s teat and avoiding crushing during the suckling process does not seem to be a process that can occur simply due to differences in body mass but is also—at least in part—due to differences in the maturation of neuromotor skills [44,50].

Similarly, it is important to mention that Muns et al. [3] have noted that the M parameter is strongly related to teat stimulation behavior (*p* < 0.001). This agrees with our results as piglets born from TPP-treated sows that exhibited high scores (2 and 3) in the M parameter performed 14–18 s more suckling of the mother’s teat (*p* < 0.0001; groups: M2 and M3 at birth and at 24 h).

In relation to the above, the fact that piglets born from TPP-treated sows that showed a low score in the tremor parameter (SH0) as well as high scores in the M and NCC parameters presented longer teat suckling periods could have influenced them to gain a higher colostrum intake and, consequently, exhibit higher weight gain at 21 days of age (>497.83 g; *p* < 0.0001; groups: M2 at 24 h and M3, NCC1, NCC2, and SH0 at birth and at 24 h). Likewise, the fact that these neonates (TPP: SH0, M, and NCC) presented better locomotion could have influenced them to have shorter time intervals in their latency to stand up, which would cause them to perform their first suckling in a faster period and increase their colostrum consumption [53,54]. Furthermore, it is important to mention that Muns et al. [3] have pointed out that the NCC parameter strongly influences piglet weight gain at weaning (*p* = 0.023), which is in agreement with our results as piglets born from TPP-treated sows that obtained high scores (1 and 2) in the NCC parameter presented higher weight gain.

Regarding the U parameter, Muns et al. [3] have shown that this parameter is correlated with body weight gain at weaning (r = 0.111, *p* = 0.084) and with piglet survival (r = 0.110, *p* = 0.063). Thus, it is considered that this parameter could be positively associated with the piglet’s ability to reach its mother’s teat and maintain suckling, therefore, promoting sow milk production as well as piglet survival and growth [3]. However, the above contrasts with our results as, in this study, it was observed that neonates from TPP-treated sows that did not demonstrate teat stimulation behavior (U0) manifested higher scores on the modified Apgar vitality scale. Furthermore, they suckled their mother’s teat for a longer time and demonstrated higher weight gain at 21 days.

Finally, it is important to note that, in terms of the tremor parameter (SH), only 1.34% of the piglets from control sows exhibited tremor behavior at birth (SH1). This could be related to the management of the piglets after birth as, at the farm where this study was conducted, the newborns of all groups went through a drying routine and had access to a heating area (boxes placed under a heat source and straw) where the piglets were confined for a short period after birth in order to minimize heat loss. In this regard, Vande Pol et al. [55] have shown that the combination of a drying-off routine and providing the newborns with a warming area has a greater effect on reducing post-natal heat loss in the newborn piglets than either technique applied separately. This is because the combination of both routines significantly reduces heat loss (during the first 20 or 45 min after birth; *p* < 0.0001) through three different routes: evaporation, convection, and radiation.

#### 4.2.2. SC

According to Muns et al. [3], the SC parameter does not have a significant effect on neonatal survival as vocalizations during the behavioral test neither increased the chances of survival of the piglets nor influenced their growth; therefore, it was observed that the SC parameter had no correlation with other explanatory variables (*p* > 0.10). In contrast, in this study, it was observed that piglets born from control sows that showed low scores in this parameter (SC0) presented significantly lower scores in the modified Apgar vitality scale (<7.23). Additionally, these piglets had 15.42–18.07 s less suckling time on the mother’s teat and exhibited lower weight gain at 21 days when compared to piglets born from TPP-treated sows (SC0). Therefore, there is a possibility that thiamine influenced weight gain at 21 days of age. It has been proposed that mechanisms regulating the body weight set point appear very early in growing breeds and that thiamine is a key regulator of this process—as reported in studies of newborn rats where neuropeptides and connections from the arcuate nucleus to the paraventricular nucleus and lateral hypothalamus develop mainly at early stages [43], suggesting that thiamine might effectively participate in the process of growth regulation and weight gain at 21 days of age in piglets born from TPP-treated sows in the SC0 group.

#### 4.2.3. UR and D

Regardless of TPP administration at the end of gestation, neonates in all groups did not exhibit defecation behavior (D0) when evaluated immediately after birth, and less than 1% of piglets in all groups exhibited urination behavior at birth (UR1). This could be related to the fact that these elimination behaviors (urination and defecation) were evaluated outside the farrowing pen. According to Nannoni et al. [56], piglets have defined areas to perform elimination behaviors and prefer to avoid elimination behaviors in the resting areas, possibly as an evolutionary function that allows them to avoid heat loss through evaporation when soiled with urine or feces; this is particularly relevant as, during the first few hours of life, neonates are very susceptible to hypothermia. Additionally, this could have been another factor—apart from the drying routine and the use of warm areas that contributed to the temperature—since, regardless of the administration of TPP, neonates in all groups that did not demonstrate urination (UR0) or defecation (D0) behavior at birth at 24 h presented a >1.7 °C increase (*p* < 0.0001) in their skin temperature from birth until they reached 24 h of age. On the other hand, it is important to mention that an effect of TPP was still observed in the modified Apgar vitality scale, teat suckling time, and weight gain as piglets born from sows treated with TPP and that did not demonstrate urination (UR) and/or defecation (D0) behavior at birth and at 24 h obtained a higher clinical vitality score (>8), suckled the teat for longer periods (>25 s), and exhibited higher weight gain at 21 days (>589 g) when compared to piglets born from control sows.

It is relevant to highlight that the results may differ if the TPP is administered directly to the neonates after birth or when the sows have finished farrowing, and also if only one dose is applied before delivery; this is because the studies [18,19,20] that found beneficial effects with this molecule describe the influence of the administration of this substance as a prophylactic treatment before the fetuses develop a hypoxic event. Therefore, the results of this study suggest that it is necessary to continue with future research to identify if the administration of a single dose before farrowing in the pregnant sow or if the administration of TPP at birth in piglets has a positive effect on the vitality and the different survival indicators of pig neonates. On the other hand, it is important to mention that in practice, the administration of TPP 24 and 12 h before farrowing could efficiently influence the energy metabolism of sows with a history of dystocia or in first-parity sows (they generally have a longer farrowing duration and the abdominal effort is usually greater than in multiparous sows). This is interesting since these alterations can decrease the growth of piglets and increase mortality before weaning. Therefore, the application of this treatment on farms could not only have an impact on the well-being of sows at farrowing but could also have an impact on farm productivity because the piglets would have a greater probability of exhibiting high vitality, greater weight gain at weaning and, therefore, increase the probability of survival in the first days after birth. However, the cost–benefit of administering this treatment in these sows would have to be analyzed since the administration of this treatment had a cost of USD 73.56 per sow.

## 5. Conclusions

Our results indicated that prophylactic administration of TPP to sows at the end of gestation decreased farrowing duration and increased the percentage of neonates, with high scores in the behavior parameters for the measurement of vitality, including movement ability (M3), udder stimulation (U1), number of circles completed around the pen (NCC2), and vocalizations (SC1) at birth and at 24 h. Similarly, the administration of TPP also increased the percentage of neonates without tremor (SH0), urination (UR0), and defecation (D0) responses at birth and 24 h, which is possibly associated with the stimulatory effect of TPP. In addition, the newborns of sows treated with TPP obtained higher scores in the parameters of movement ability (M3) and number of completed circles around the pen (NCC1 and NCC2), as well as low scores in the parameters of udder stimulation (U0), vocalizations (SH0), shivering (SH0), urination (UR0), and defecation (D0). Furthermore, they presented higher clinical vitality scores, suckled the teat for longer periods, and obtained a higher weight gain at 21 days, likely as a result of the abovementioned protective effect of TPP.

Although thiamine is a vitamin administered in the diets of pregnant sows, it is found in small quantities as free thiamine and not as phosphorylated thiamine. In order for it to change to its phosphorylated form, the sow has to expend energy to phosphorylate free thiamine. In practice, the administration of TPP is intended to efficiently influence energy metabolism hours before farrowing by acting as a coenzyme in several enzymes involved in the production of ATP. This is because during farrowing, greater energy extraction by the uterus is used to produce efficient uterine contractions and for abdominal effort since these, being energy-dependent, can compromise the energy status of the sow. For this reason, the administration of TPP is recommended 24 and 12 h before farrowing in sows with a history of dystocia or in first-parity sows (they generally have a longer farrowing duration and the abdominal effort is usually greater than in multiparous sows).

On the other hand, in practice, the application of this treatment might not only reduce the probability of sows presenting these problems but their offspring may also have a greater probability of exhibiting high vitality as well as a greater weight gain at weaning and, therefore, increase the probability of surviving in the first days after birth.

At the economic level, the total cost of treatment would be USD 73.56 per sow, which could be viable considering that hyperprolific sows—such as those found in intensive production—have a total of 13.8 ± 0.2 piglets born alive per litter (range: 0–22 live-born piglets); moreover, taking into account hyperprolific sows with 2 to 5 parturitions—such as those used in this experiment—the number of live-born piglets is 15.2 ± 0.1.

Although the cost of treatment seems high, it is important to mention that this study is focused on intensive production and not on backyard farms, which should probably be provided with other options with prior scientific validation. In addition, this was one of the first studies to evaluate the effect of TPP as a prophylactic treatment on different indicators of vitality and survival so that the dose can be adjusted at a later stage and the same effects can be possibly achieved with a lower dose, which will reduce treatment costs at the production level.

## Figures and Tables

**Table 1:** Description of behavioral variables evaluated to establish vitality of piglets according to Muns et al. [3], Tuchscherer et al. [32], and Lürzel et al. [33].

Variable
Movement capacity (M) M0: unable to keep voluntary position M1: able to keep voluntary position but unable to move (unable to turn its body axis > 90° from its initial orientation) M2: moving “slowly” (able to turn its body axis > 90° from its initial orientation within 30 s) M3: moving “fast” (able to turn its body axis > 90° from its initial orientation within 15 s)
Udder stimulation (U) U0: shows no head movements emulating udder stimulation movements or searching behavior within 30 s U1: shows head movements emulating udder stimulation movements or searching behavior within 30 s
Number of completed circles around enclosure (NCC) NCC0: not able to turn its body axis 360° from its initial orientation nor able to walk along the limits of the bucket NCC1: able to turn its body axis 360° from its initial orientation or walk along the limits of the bucket once within 30 s NCC2: able to turn its body axis 360° from its initial orientation or walk along the limits of the bucket at least twice within 30 s
Screaming (SC) SC0: piglet does not scream during manipulation/observation time SC1: piglet does scream during manipulation/observation time
Shivering (SH) SH0: no tremor is present SH1: shivering—piglets lay separately on the floor or stand and display rapid, synchronous muscle contractions, frequently accompanied by piloerection
Urination (UR) UR0: the animal does not release urine UR1: the animal releases urine
Defecation (D) D0: the animal does not release feces D1: the animal releases feces

**Table 2:** Reproductive variables of sows treated with and without thiamine pyrophosphate.

	Sows Without TPP (*n* = 6)	Sows with TPP (*n* = 9)	*p*-Value
Duration of farrowing (min)	198.66 ± 11.48	150.11 ± 9.37	0.0060
Total number of piglets born per litter	15.33 ± 3.14	16.77 ± 2.04	0.2974
Number of piglets born alive per litter	14.33 ± 2.50	16.44 ± 1.87	0.0839
Number of stillborn piglets per litter	0.8 ± 0.89	0.375 ± 0.5	0.2770
Number of piglets at weaning per litter	12.16 ± 1.22	11.77 ± 0.99	0.8091

Data are presented as mean ± standard error (SE); *p* < 0.05; *n* = number of sows treated with and without TPP.

**Table 3:** Percentage of observation time spent in behaviors by piglets from control and TPP sows.

Parameter	Control (*n* = 76)		TPP (*n* = 73)	
Movement capacity (M)	Birth (Frequency/%)	24 h (Frequency/%)	Birth (Frequency/%)	24 h (Frequency/%)
0	0/0	2/2.64%	3/4.10%	0/0
1	9/11.84%	3/3.94%	3/4.10%	0/0
2	24/31.58%	34/44.74%	16/21.91%	16/21.91%
3	43/56.58%	37/48.68%	51/69.89%	57/78.09%
	76/100%	76/100%	73/100%	73/100%
Udder stimulation (U)				
0	23/30.26%	33/43.43%	15/20.54%	22/30.13%
1	53/69.74%	43/56.57%	58/79.46%	51/69.87%
	76/100%	76/100%	73/100%	73/100%
Number of completed circles around enclosure (NCC)				
0	8/10.53%	5/6.57%	6/8.22%	1/1.37%
1	39/51.31%	45/59.21%	21/28.76%	34/46.57%
2	29/38.16%	26/34.22%	46/63.02%	38/52.06%
	76/100%	76/100%	73/100%	73/100%
Screaming (SC)				
0	34/44.74%	34/44.74%	25/34.25%	29/39.73%
1	42/55.26%	42/55.26%	48/65.75%	44/60.27%
	76/100%	76/100	73/100%	73/100%
Shivering (SH)				
0	74/97.36%	76/100%	73/100%	73/100%
1	2/2.64%	0/0	0/0	0/0
	76/100%	76/100%	73/100%	73/100%
Urination (UR)				
0	75/98.68%	70/92.10%	72/98.64%	62/84.94%
1	1/1.32%	6/7.90%	1/1.36%	11/15.06%
	76/100%	76/100%	73/100%	73/100%
Defecation (D)				
0	76/100%	73/96.05%	73/100%	73/100%
1	0/0	3/3.95%	0/0	0/0
	76/100%	76/100%	73/100%	73/100%

**Table 4:** Effect of thiamine pyrophosphate on expulsion interval, modified Apgar vitality, SpO_2_, suckling time, weight, and temperature according to the movement capacity parameter (M) evaluated at birth and at 24 h in neonatal piglets.

			Piglets Without TPP (*n* = 76)	Piglets With TPP (*n* = 73)	
Interval of explusion (min)					*p*-value
0		Birth	-	10.00 ± 3.43	0.7369
		24 h	12.00 ± 4.20	-	
1		Birth	20.85 ± 7.43 ^a^	20.85 ± 7.20 ^a^	0.2880
		24 h	31.33 ± 11.35 ^a^	-	
2		Birth	22.00 ± 4.16 ^a^	11.85 ± 4.58 ^a^	0.3269
		24 h	19.53 ± 3.36 ^a^	14.46 ± 4.43 ^a^	
3		Birth	14.54 ± 1.77 ^a^	15.65 ± 1.69 ^a^	0.9357
		24 h	14.67 ± 1.96 ^a^	14.18 ± 1.62 ^a^	
Vitality score at birth (modified Apgar)					
0		Birth	-	7.68 ± 0.49 ^a^	0.5438
		24 h	7.15 ± 0.60 ^a^	-	
1		Birth	5.39 ± 0.36 ^ab^	7.03 ± 0.64 ^a^	<0.0357 *
		24 h	4.37 ± 0.64 ^b^	-	
2		Birth	6.93 ± 0.29 ^a^	7.84 ± 0.30 ^a^	0.0606
		24 h	7.06 ± 0.22 ^a^	7.73 ± 0.30 ^a^	
3		Birth	7.84 ± 0.17 ^b^	8.50 ± 0.17 ^a^	<0.0049 *
		24 h	7.75 ± 0.20 ^b^	8.39 ± 0.16 ^ab^	
SpO_2_ (%) at birth					
0		Birth	-	89.50 ± 5.50	0.9854
		24 h	89.50 ± 5.50	-	
1		Birth	92.00 ± 4.04 ^a^	91.00 ± 4.04 ^a^	0.1496
		24 h	81.33 ± 2.33 ^a^	-	
2		Birth	87.83 ± 2.14 ^a^	85.00 ± 2.27 ^ab^	0.0394 *
		24 h	84.500 ± 1.61 ^ab^	78.75 ± 2.27 ^b^	
3		Birth	83.23 ± 1.71 ^a^	82.98 ± 1.62 ^a^	0.9854
		24 h	83.91 ± 1.90 ^a^	83.21 ± 1.54 ^a^	
Suckling time (s)					
0		Birth	-	24.33 ± 4.40 ^a^	0.3587
		24 h	19.00 ± 5.39 ^a^	-	
1		Birth	13.43 ± 2.14 ^a^	11.33 ± 3.92 ^a^	0.8531
		24 h	14.33 ± 3.92 ^a^	-	
2		Birth	11.38 ± 2.97 ^b^	29.70 ± 3.15 ^a^	<0.0001 *
		24 h	11.03 ± 2.34 ^b^	25.18 ± 3.15 ^a^	
3		Birth	13.23 ± 1.78 ^b^	27.27 ± 1.69 ^a^	<0.0001 *
		24 h	13.18 ± 1.98 ^b^	25.67 ± 1.61 ^a^	
Weight (g)					
0	Birth	Weight at birth	-	1593.33 ± 141.29 ^c^	<0.0001 *
		Weight at 21 days	-	5886.67 ± 141.29 ^a^	
	24 h	Weight at birth	1440 ± 173.04 ^c^	-	
		Weight at 21 days	4090 ± 173.04 ^b^	-	
1	Birth	Weight at birth	1581.11 ± 114.27 ^b^	1046.67 ± 197.93 ^b^	<0.0001 *
		Weight at 21 days	5513.33 ± 114.27 ^a^	5966.67 ± 197.93 ^a^	
	24 h	Weight at birth	1573.33 ± 197.93 ^b^	-	
		Weight at 21 days	5856.67 ± 197.93 ^a^	-	
2	Birth	Weight at birth	1671.67 ± 92.79 ^c^	1716.25 ± 97.77 ^c^	<0.0001 *
		Weight at 21 days	5614.44 ± 92.17 ^ab^	5960.00 ± 97.77 ^a^	
	24 h	Weight at birth	1559.31 ± 72.62 ^c^	1398.13 ± 97.77 ^c^	
		Weight at 21 days	5497.93 ± 72.62 ^b^	6000.00 ± 97.77 ^a^	
3	Birth	Weight at birth	1537.85 ± 72.68 ^c^	1543.14 ± 69.03 ^c^	<0.0001 *
		Weight at 21 days	5233.64 ± 74.32 ^b^	6024.00 ± 69.72 ^a^	
	24 h	Weight at birth	1602.16 ± 81.04 ^c^	1600.89 ± 65.88 ^c^	
		Weight at 21 days	5349.44 ± 82.16 ^b^	5998.18 ± 66.47 ^a^	
Temperature (°C)					
0	Birth	Temperature at birth	-	36.16 ± 0.31 ^b^	<0.0013 *
		Temperature at 24 h	-	38.36 ± 0.31 ^a^	
	24 h	Temperature at birth	35.40 ± 0.38 ^b^	-	
		Temperature at 24 h	38.80 ± 0.38 ^a^	-	
1	Birth	Temperature at birth	36.13 ± 0.16 ^c^	36.86 ± 0.28 ^bc^	<0.0001 *
		Temperature at 24 h	37.70 ± 0.16 ^b^	39.23 ± 0.28 ^a^	
	24 h	Temperature at birth	36.56 ± 0.28 ^c^	-	
		Temperature at 24 h	37.96 ± 0.28 ^b^	-	
2	Birth	Temperature at birth	36.28 ± 0.12 ^c^	36.23 ± 0.13 ^c^	<0.0001 *
		Temperature at 24 h	37.74 ± 0.12 ^ab^	38.09 ± 0.13 ^ab^	
	24 h	Temperature at birth	36.30 ± 0.10 ^c^	36.31 ± 0.13 ^c^	
		Temperature at 24 h	37.77 ± 0.10 ^b^	38.31 ± 0.13 ^a^	
3	Birth	Temperature at birth	36.18 ± 0.09 ^b^	36.25 ± 0.09 ^b^	<0.0001 *
		Temperature at 24 h	38.15 ± 0.09 ^a^	38.20 ± 0.09 ^a^	
	24 h	Temperature at birth	36.12 ± 0.10 ^b^	36.25 ± 0.08 ^b^	
		Temperature at 24 h	38.11 ± 0.10 ^a^	38.20 ± 0.08 ^a^	

Data are presented as mean ± standard error (SE); different letters indicate statistical differences between groups with *p* < 0.05; *n* = number of newborn piglets sampled. * = Indicates statistical differences with *p* < 0.05.

**Table 5:** Effect of thiamine pyrophosphate on expulsion interval, modified Apgar vitality, SpO_2_, suckling time, weight, and temperature according to the udder stimulation (U) parameter evaluated at birth and at 24 h in neonatal piglets.

			Piglets Without TPP (*n* = 76)	Piglets with TPP (*n* = 73)	
Interval of expulsion (min)					*p*-value
0		Birth	19.58 ± 3.57 ^ab^	8.21 ± 3.93 ^c^	<0.0222 *
		24 h	20.34 ± 2.88 ^a^	9.83 ± 3.47 ^bc^	
1		Birth	16.28 ± 1.87 ^a^	15.67 ± 1.8 ^a^	0.9869
		24 h	15.28 ± 2.09 ^a^	15.93 ± 1.91 ^a^	
Vitality score at birth (modified Apgar)					
0		Birth	6.51 ± 0.28 ^b^	7.66 ± 0.33 ^ab^	<0.0001 *
		24 h	6.80 ± 0.24 ^b^	8.32 ± 0.27 ^a^	
1		Birth	7.62 ± 0.17 ^b^	8.41 ± 0.16 ^a^	<0.0021 *
		24 h	7.64 ± 0.19 ^b^	8.21 ± 0.18 ^ab^	
SpO_2_ (%) at birth					
0		Birth	82.70 ± 2.57 ^a^	80.66 ± 2.97 ^a^	0.9511
		24 h	81.53 ± 2.17 ^a^	80.95 ± 2.45 ^a^	
1		Birth	84.20 ± 1.52 ^a^	82.75 ± 1.45 ^a^	0.4536
		24 h	86.02 ± 1.68 ^a^	82.92 ± 1.55 ^a^	
Suckling time (s)					
0		Birth	11.25 ± 1.51 ^b^	21.00 ± 1.74 ^a^	<0.0001 *
		24 h	10.17 ± 1.27 ^b^	17.68 ± 1.44 ^a^	
1		Birth	15.54 ± 1.72 ^b^	26.87 ± 1.65 ^a^	<0.0001 *
		24 h	15.41 ± 1.91 ^b^	25.60 ± 1.77 ^a^	
Weight (g)					
0	Birth	Weight at birth	1593.00 ± 97.77 ^c^	1539.33 ± 112.90 ^c^	<0.0001 *
		Weight at 21 days	5423.50 ± 97.77 ^b^	5921.33 ± 112.90 ^a^	
	24 h	Weight at birth	1541.43 ± 82.63 ^c^	1517.27 ± 93.22 ^c^	
		Weight at 21 days	5458.21 ± 82.63 ^b^	6045.45 ± 93.22 ^a^	
1	Birth	Weight at birth	1569.83 ± 66.40 ^c^	1568.79 ± 63.48 ^c^	<0.0001 *
		Weight at 21 days	5342.94 ± 67.69 ^b^	6019.30 ± 64.03 ^a^	
	24 h	Weight at birth	1603.26 ± 73.72 ^c^	1572.80 ± 68.37 ^c^	
		Weight at 21 days	5355.71 ± 74.60 ^b^	5977.55 ± 69.06 ^a^	
Temperature (°C)					
0	Birth	Temperature at birth	36.30 ± 0.13 ^c^	36.30 ± 0.15 ^c^	<0.0001 *
		Temperature at 24 h	37.81 ± 0.13 ^b^	38.24 ± 0.15 ^ab^	
	24 h	Temperature at birth	36.29 ± 0.11 ^c^	36.22 ± 0.12 ^c^	
		Temperature at 24 h	37.86 ± 0.11 ^b^	38.41 ± 0.12 ^a^	
1	Birth	Temperature at birth	36.16 ± 0.08 ^b^	36.23 ± 0.08 ^b^	<0.0001 *
		Temperature at 24 h	38.06 ± 0.08 ^a^	38.21 ± 0.08 ^a^	
	24 h	Temperature at birth	36.12 ± 0.09 ^b^	36.29 ± 0.08 ^b^	
		Temperature at 24 h	38.06 ± 0.09 ^a^	38.14 ± 0.08 ^a^	

Data are presented as mean ± standard error (SE); different letters indicate statistical differences between groups with *p* < 0.05; *n* = number of newborn piglets sampled. * = Indicates statistical differences with *p* < 0.05.

**Table 6:** Effect of thiamine pyrophosphate on expulsion interval, modified Apgar vitality, SpO_2_, suckling time, weight, and temperature according to the number of completed circles around enclosure (NCC) parameter evaluated at birth and at 24 h in neonatal piglets.

			Piglets Without TPP (*n* = 76)	Piglets with TPP (*n* = 73)	
Interval of explusion (min)					*p*-value
0		Birth	9.33 ± 3.75 ^a^	6.00 ± 3.75 ^a^	0.1768
		24 h	18.75 ± 4.59 ^a^	1.00 ± 9.19 ^a^	
1		Birth	18.47 ± 2.56 ^a^	11.94 ± 3.43 ^a^	0.2783
		24 h	17.77 ± 2.49 ^a^	13.16 ± 2.73 ^a^	
2		Birth	16.81 ± 2.45 ^a^	16.07 ± 1.99 ^a^	0.9633
		24 h	16.40 ± 2.55 ^a^	15.17 ± 2.15 ^a^	
Vitality score at birth (modified Apgar)					
0		Birth	5.72 ± 0.71 ^a^	7.35 ± 0.71 ^a^	0.1881
		24 h	5.91 ± 0.87 ^a^	-	
1		Birth	7.14 ± 0.21 ^bc^	8.06 ± 0.28 ^ab^	<0.0008 *
		24 h	7.13 ± 0.20 ^c^	8.15 ± 0.22 ^a^	
2		Birth	7.87 ± 0.22 ^ab^	8.41 ± 0.18 ^a^	<0.0400 *
		24 h	7.74 ± 0.22 ^b^	8.40 ± 0.20 ^a^	
SpO_2_ (%) at birth					
0		Birth	89.50 ± 10.86 ^a^	69.66 ± 6.27 ^a^	0.4405
		24 h	86.00 ± 7.68 ^a^	70.00 ± 15.36 ^a^	
1		Birth	83.71 ± 1.65 ^a^	86.66 ± 2.23 ^a^	0.4888
		24 h	83.12 ± 1.59 ^a^	82.41 ± 1.75 ^a^	
2		Birth	85.03 ± 2.15 ^a^	82.00 ± 1.70 ^a^	0.4568
		24 h	85.76 ± 2.27 ^a^	82.37 ± 1.90 ^a^	
Suckling time (s)					
0		Birth	14.83 ± 5.72 ^a^	15.00 ± 5.72 ^a^	0.3045
		24 h	-		
1		Birth	13.26 ± 2.02 ^b^	28.47 ± 2.73 ^a^	<0.0001 *
		24 h	12.60 ± 1.94 ^b^	24.52 ± 2.14 ^a^	
2		Birth	14.03 ± 2.41 ^b^	26.52 ± 1.91 ^a^	<0.0001 *
		24 h	14.34 ± 2.55 ^b^	24.02 ± 2.13 ^a^	
Weight (g)					
0	Birth	Weight at birth	1928.33 ± 189.35 ^c^	1320.00 ± 189.35 ^c^	<0.0001 *
		Weight at 21 days	5460.00 ± 189.35 ^ab^	5926.67 ± 189.35 ^a^	
	24 h	Weight at birth	1450.00 ± 231.90 ^c^	1320.00 ± 463.80 ^c^	
		Weight at 21 days	4932.50 ± 23.90 ^b^	6250.00 ± 463.80 ^ab^	
1	Birth	Weight at birth	1593.16 ± 73.14 ^c^	1699.05 ± 98.39 ^c^	<0.0001 *
		Weight at 21 days	5481.32 ± 73.14 ^b^	6050.50 ± 100.82 ^a^	
	24 h	Weight at birth	1602.44 ± 70.41 ^c^	1555.59 ± 77.32 ^c^	
		Weight at 21 days	5515.85 ± 70.41 ^b^	5993.33 ± 78.49 ^a^	
2	Birth	Weight at birth	1481.07 ± 88.72 ^c^	1532.17 ± 70.44 ^c^	<0.0001 *
		Weight at 21 days	5181.85 ± 91.95 ^b^	5990.22 ± 70.44 ^a^	
	24 h	Weight at birth	1561.54 ± 93.70 ^c^	1562.43 ± 78.55 ^c^	
		Weight at 21 days	5275.60 ± 95.56 ^b^	5996.49 ± 78.55 ^a^	
Temperature (°C)					
0	Birth	Temperature at birth	36.06 ± 0.26 ^c^	36.51 ± 0.26 ^bc^	<0.0001 *
		Temperature at 24 h	37.71 ± 0.26 ^ab^	38.80 ± 0.26 ^a^	
	24 h	Temperature at birth	36.07 ± 0.32 ^c^	37.00 ± 0.64 ^abc^	
		Temperature at 24 h	38.47 ± 0.32 ^a^	39.00 ± 0.64 ^a^	
1	Birth	Temperature at birth	36.24 ± 0.09 ^b^	36.23 ± 0.12 ^b^	<0.0001 *
		Temperature at 24 h	37.84 ± 0.09 ^a^	38.09 ± 0.13 ^a^	
	24 h	Temperature at birth	36.24 ± 0.09 ^b^	36.21 ± 0.10 ^b^	
		Temperature at 24 h	37.90 ± 0.09 ^a^	38.22 ± 0.10 ^a^	
2	Birth	Temperature at birth	36.17 ± 0.11 ^b^	36.25 ± 0.09 ^b^	<0.0001 *
		Temperature at 24 h	38.25 ± 0.12 ^a^	38.18 ± 0.09 ^a^	
	24 h	Temperature at birth	36.13 ± 0.12 ^b^	36.30 ± 0.10 ^b^	
		Temperature at 24 h	38.04 ± 0.12 ^a^	38.20 ± 0.10 ^a^	

Data are presented as mean ± standard error (SE); different letters indicate statistical differences between groups with *p* < 0.05; *n* = number of newborn piglets sampled. * = Indicates statistical differences with *p* < 0.05.

**Table 7:** Effect of thiamine pyrophosphate on expulsion interval, modified Apgar vitality, SpO_2_, suckling time, weight, and temperature according to the screaming parameter (SC) evaluated at birth and at 24 h in neonatal piglets.

			Piglets Without TPP (*n* = 76)	Piglets with TPP (*n* = 73)	
Interval of expulsion (min)					*p*-value
0		Birth	18.18 ± 3.07 ^a^	12.30 ± 3.13 ^a^	0.2293
		24 h	21.43 ± 3.13 ^a^	17.95 ± 3.32 ^a^	
1		Birth	16.41 ± 1.90 ^a^	15.25 ± 1.88 ^a^	0.4408
		24 h	14.56 ± 1.90 ^a^	12.21 ± 1.83 ^a^	
Vitality score at birth (modified Apgar)					
0		Birth	6.72 ± 0.21 ^b^	8.16 ± 0.23 ^a^	<0.0001 *
		24 h	7.23 ± 0.21 ^b^	8.48 ± 0.23 ^a^	
1		Birth	7.77 ± 0.20 ^ab^	8.35 ± 0.20 ^a^	<0.0189 *
		24 h	7.46 ± 0.20 ^b^	8.08 ± 0.20 ^ab^	
SpO_2_ (%) at birth					
0		Birth	82.35 ± 1.82 ^a^	81.17 ± 1.88 ^a^	0.3926
		24 h	84.86 ± 1.88 ^a^	85.00 ± 1.91 ^a^	
1		Birth	84.85 ± 1.83 ^a^	83.09 ± 1.78 ^a^	0.3560
		24 h	83.83 ± 1.83 ^a^	80.45 ± 1.78 ^a^	
Suckling time (s)					
0		Birth	12.54 ± 2.51 ^b^	27.96 ± 2.59 ^a^	<0.0001 *
		24 h	12.68 ± 2.59 ^b^	30.75 ± 2.64 ^a^	
1		Birth	13.85 ± 1.75 ^b^	25.52 ± 1.71 ^a^	<0.0001 *
		24 h	13.88 ± 1.75 ^b^	21.20 ± 1.71 ^a^	
Weight (g)					
0	Birth	Weight at birth	1545.19 ± 81.66 ^c^	1617.59 ± 84.43 ^c^	<0.0001 *
		Weight at 21 days	5424.33 ± 83.01 ^b^	6002.76 ± 84.43 ^a^	
	24 h	Weight at birth	1527.59 ± 84.43 ^c^	1708.21 ± 85.92 ^c^	
		Weight at 21 days	5367.93 ± 84.43 ^b^	6089.64 ± 85.92 ^a^	
1	Birth	Weight at birth	1599.05 ± 73.54 ^c^	1526.59 ± 71.85 ^c^	<0.0001 *
		Weight at 21 days	5322.68 ± 74.43 ^b^	6000.93 ± 72.68 ^a^	
	24 h	Weight at birth	1614.29 ± 73.54 ^c^	1458.86 ± 71.85 ^c^	
		Weight at 21 days	5417.07 ± 74.43 ^b^	5939.30 ± 72.68 ^a^	
Temperature (°C)					
0	Birth	Temperature at birth	36.30 ± 0.11 ^b^	36.33 ± 0.11 ^b^	<0.0001 *
		Temperature at 24 h	38.07 ± 0.11 ^a^	38.09 ± 0.11 ^a^	
	24 h	Temperature at birth	36.15 ± 0.11 ^b^	36.17 ± 0.11 ^b^	
		Temperature at 24 h	37.99 ± 0.11 ^a^	37.93 ± 0.11 ^a^	
1	Birth	Temperature at birth	36.12 ± 0.09 ^c^	36.22 ± 0.09 ^c^	<0.0001 *
		Temperature at 24 h	37.94 ± 0.09 ^b^	38.09 ± 0.09 ^ab^	
	24 h	Temperature at birth	36.22 ± 0.09 ^c^	36.33 ± 0.09 ^c^	
		Temperature at 24 h	37.98 ± 0.09 ^b^	38.39 ± 0.09 ^a^	

Data are presented as mean ± standard error (SE); different letters indicate statistical differences between groups with *p* < 0.05; *n* = number of newborn piglets sampled. * = Indicates statistical differences with *p* < 0.05.

**Table 8:** Effect of thiamine pyrophosphate on expulsion interval, modified Apgar vitality, SpO_2_, suckling time, weight, and temperature according to the shivering parameter (SH) evaluated at birth and at 24 h in neonatal piglets.

			Piglets Without TPP (*n* = 76)	Piglets with TPP (*n* = 73)	
Interval of expulsion (min)					*p*-value
0		Birth	17.13 ± 1.69 ^a^	14.62 ± 1.76 ^a^	0.4476
		24 h	17.30 ± 1.70 ^a^	14.24 ± 1.70 ^a^	
1		Birth	7.60 ± 2.82	-	-
		24 h	-	-	
Vitality score at birth (modified Apgar)					
0		Birth	7.31 ± 0.15 ^b^	8.31 ± 0.16 ^a^	<0.0001 *
		24 h	7.31 ± 0.15 ^b^	8.24 ± 0.15 ^a^	
1		Birth	7.47 ± 0.35	-	-
		24 h	-	-	
SpO_2_ (%) at birth					
0		Birth	83.79 ± 1.27 ^a^	83.26 ± 1.31 ^a^	0.7072
		24 h	84.25 ± 1.28 ^a^	82.22 ± 1.27 ^a^	
1		Birth	69.60 ± 9.97	-	-
		24 h	-	-	
Suckling time (s)					
0		Birth	13.67 ± 1.42 ^b^	26.23 ± 1.47 ^a^	<0.0001 *
		24 h	13.43 ± 1.44 ^b^	25.02 ± 1.43 ^a^	
1		Birth	14.60 ± 2.56	-	-
		24 h	-	-	
Weight (g)					
0	Birth	Weight at birth	1576.18 ± 55.09 ^c^	1576.18 ± 57.07 ^c^	<0.0001 *
		Weight at 21 days	5365.63 ± 55.86 ^b^	6004.63 ± 57.50 ^a^	
	24 h	Weight at birth	1578.87 ± 55.86 ^c^	1555.83 ± 55.47 ^c^	
		Weight at 21 days	5396.71 ± 56.25 ^b^	5998.59 ± 55.86 ^a^	
1	Birth	Weight at birth	1380 ± 140.08 ^a^	-	<0.0001 *
		Weight at 21 days	5962 ± 140.08 ^b^	-	
	24 h	Weight at birth	-	-	
		Weight at 21 days	-	-	
Temperature (°C)					
0	Birth	Temperature at birth	36.20 ± 0.07 ^b^	36.25 ± 0.07 ^b^	<0.0001 *
		Temperature at 24 h	37.99 ± 0.07 ^a^	38.16 ± 0.07 ^a^	
	24 h	Temperature at birth	36.19 ± 0.07 ^b^	36.27 ± 0.07 ^b^	
		Temperature at 24 h	37.98 ± 0.07 ^a^	38.21 ± 0.07 ^a^	
1	Birth	Temperature at birth	36.46 ± 0.32 ^a^	-	<0.0008 *
		Temperature at 24 h	38.86 ± 0.32 ^b^	-	
	24 h	Temperature at birth	-	-	
		Temperature at 24 h	-	-	

Data are presented as mean ± standard error (SE); different letters indicate statistical differences between groups with *p* < 0.05; *n* = number of newborn piglets sampled. * = Indicates statistical differences with *p* < 0.05.

**Table 9:** Effect of thiamine pyrophosphate on expulsion interval, modified Apgar vitality, SpO_2_, suckling time, weight, and temperature according to urination parameter (UR) evaluated at birth and at 24 h in neonatal piglets.

			Piglets Without TPP (*n* = 76)	Piglets with TPP (*n* = 73)	
Interval of expulsion (min)					*p*-value
0		Birth	16.96 ± 1.72 ^a^	14.29 ± 1.72 ^a^	0.5014
		24 h	17.49 ± 1.77 ^a^	14.87 ± 1.86 ^a^	
1		Birth	28.00 ± 10.00 ^a^	1.00 ± 10.99 ^a^	0.3952
		24 h	12.00 ± 4. 91 ^a^	10.80 ± 3.47 ^a^	
Vitality score at birth (modified Apgar)					
0		Birth	7.29 ± 0.15 ^b^	8.28 ± 0.15 ^a^	<0.0001 *
		24 h	7.28 ± 0.16 ^b^	8.27 ± 0.17 ^a^	
1		Birth	8.75 ± 1.18 ^a^	6.80 ± 1.18 ^a^	<0.4999
		24 h	7.47 ± 0.48 ^a^	8.10 ± 0.35 ^a^	
SpO_2_ (%) at birth					
0		Birth	83.70 ± 1.34 ^a^	82.32 ± 1.33 ^a^	0.4800
		24 h	84.64 ± 1.41 ^a^	81.83 ± 1.45 ^a^	
1		Birth	90.00 ± 7.85 ^a^	89.00 ± 7.85 ^a^	0.6331
		24 h	81.42 ± 2.96 ^a^	84.36 ± 2.36 ^a^	
Suckling time (s)					
0		Birth	13.69 ± 1.51 ^b^	25.51 ± 1.51 ^a^	< 0.0001 *
		24 h	13.38 ± 1.57 ^b^	25.62 ± 1.64 ^a^	
1		Birth	45.00 ± 8.97 ^a^	37.00 ± 8.97 ^ab^	< 0.0069 *
		24 h	11.16 ± 3.66 ^c^	22.72 ± 2.70 ^b^	
Weight (g)					
0	Birth	Weight at birth	1579.74 ± 55.67 ^c^	1560.28 ± 55.67 ^c^	<0.0001 *
		Weight at 21 days	5378.43 ± 56.46 ^b^	6004.51 ± 56.06 ^a^	
	24 h	Weight at birth	1584.18 ± 57.71 ^c^	1565.41 ± 60.48 ^c^	
		Weight at 21 days	5385.76± 58.14 ^b^	5975.74 ± 60.48 ^a^	
1	Birth	Weight at birth	1320.00 ± 363.85 ^c^	1740.00 ± 363.85 ^c^	<0.0001 *
		Weight at 21 days	4470.00 ± 363.85 ^b^	5800.00 ± 363.85 ^ab^	
	24 h	Weight at birth	1495.00 ± 148.54 ^c^	1502.00 ± 109.70 ^c^	
		Weight at 21 days	5551.67 ± 148.54 ^ab^	6138.00 ± 115.06 ^a^	
Temperature (°C)					
0	Birth	Temperature at birth	36.19 ± 0.72 ^b^	36.26 ± 0.07 ^b^	<0.0001 *
		Temperature at 24 h	37.97 ± 0.07 ^a^	38.21 ± 0.07 ^a^	
	24 h	Temperature at birth	36.20 ± 0.07 ^b^	36.27 ± 0.07 ^b^	
		Temperature at 24 h	37.99 ± 0.07 ^a^	38.19 ± 0.07 ^a^	
1	Birth	Temperature at birth	36.60 ± 0.72 ^ab^	36.60 ± 0.72 ^ab^	<0.0001 *
		Temperature at 24 h	39.20 ± 0.72 ^a^	38.20 ± 0.72 ^ab^	
	24 h	Temperature at birth	36.10 ± 0.29 ^b^	36.25 ± 0.21 ^b^	
		Temperature at 24 h	37.78 ± 0.29 ^a^	38.36 ± 0.23 ^a^	

Data are presented as mean ± standard error (SE); different letters indicate statistical differences between groups with *p* < 0.05; *n* = number of newborn piglets sampled. * = Indicates statistical differences with *p* < 0.05.

**Table 10:** Effect of thiamine pyrophosphate on expulsion interval, modified Apgar vitality, SpO_2_, suckling time, weight, and temperature according to defecation parameter (D) evaluated at birth and at 24 h in neonatal piglets.

			Piglets Without TPP (*n* = 76)	Piglets with TPP (*n* = 73)	
Interval of expulsion (min)					*p*-value
0		Birth	17.33 ± 1.66 ^a^	14.09 ± 1.70 ^a^	0.3108
		24 h	17.40 ± 1.75 ^a^	14.11 ± 1.75 ^a^	
1		Birth	-	-	-
		24 h	15.33 ± 5.48	-	
Vitality score at birth (modified Apgar)					
0		Birth	7.31 ± 0.15 ^b^	8.26 ± 0.15 ^a^	<0.0001 *
		24 h	7.34 ± 0.16 ^b^	8.24 ± 0.15 ^a^	
1		Birth	-	-	-
		24 h	6.66 ± 0.90	-	
SpO_2_ (%) at birth					
0		Birth	83.98 ± 1. 34 ^a^	78.76 ± 1.68 ^a^	0.0538
		24 h	84.27 ± 1.39 ^a^	82.25 ± 1.37 ^a^	
1		Birth	-	-	-
		24 h	79.33 ± 1.76	-	
Suckling time (s)					
0		Birth	14.37 ± 1.49 ^b^	25.95 ± 1.50 ^a^	< 0.0001 *
		24 h	13.41 ± 1.55 ^b^	25.36 ± 1.54 ^a^	
1		Birth	-	-	-
		24 h	10.33 ± 3.84	-	
Weight (g)					
0	Birth	Weight at birth	1576.77 ± 54.51 ^c^	1562.74 ± 54.88 ^c^	<0.0001 *
		Weight at 21 days	5365.69 ± 55.26 ^b^	6001.76 ± 55.26 ^a^	
	24 h	Weight at birth	1572.65 ± 56.87 ^c^	1539.71 ± 56.45 ^c^	
		Weight at 21 days	5389.85 ± 57.29 ^b^	5988.24 ± 56.87 ^a^	
1	Birth	Weight at birth	-	-	<0.0005 *
		Weight at 21 days	-	-	
	24 h	Weight at birth	1720.00 ± 261.18 ^a^	-	
		Weight at 21 days	5550.00 ± 261.18 ^b^	-	
Temperature (°C)					
0	Birth	Temperature at birth	36.20 ± 0.07 ^b^	36.26 ± 0.07 ^b^	<0.0001 *
		Temperature at 24 h	38.01 ± 0.07 ^a^	38.21 ± 0.07 ^a^	
	24 h	Temperature at birth	36.19 ± 0.07 ^b^	36.30 ± 0.07 ^b^	
		Temperature at 24 h	37.99 ± 0.07 ^a^	38.24 ± 0.07 ^a^	
		Temperature at birth	-	-	<0.0074 *
1	Birth	Temperature at 24 h	-	-	
	24 h	Temperature at birth	36.23 ± 0.22 ^a^	-	
		Temperature at 24 h	37.80 ± 0.22 ^b^	-	

Data are presented as mean ± standard error (SE); different letters indicate statistical differences between groups with *p* < 0.05; *n* = number of newborn piglets sampled. * = Indicates statistical differences with *p* < 0.05.

## Data Availability

The data presented in this study are available in this paper.

## Abbreviations

The following abbreviations are used in this manuscript:

ATP	Adenosine triphosphate
D	Defecation
M	Movement capacity
NADPH	Nicotinamide adenine dinucleotide phosphate
NCC	Number of completed circles around enclosure
TPP	Thiamine pyrophosphate
RNS	Reactive nitrogen species
ROS	Reactive oxygen species
SC	Screaming
SH	Shivering
U	Udder stimulation
UR	Urination
